# *ATCodeR*: a dictionary-based R-tool to standardize medication free-text

**DOI:** 10.1038/s41598-025-97150-9

**Published:** 2025-04-10

**Authors:** Isabel Schnorr, Stefanie Andreas, Linnea Schumann, Svenja Hahn, Jörg Janne Vehreschild, Daniel Maier

**Affiliations:** 1 Faculty of Medicine, Institute for Digital Medicine and Clinical Data Sciences, Goethe University Frankfurt, Frankfurt, Germany; 2 Medical Department 2 (Hematology/Oncology and Infectious Diseases), Center for Internal Medicine, University Hospital, Goethe University Frankfurt, Frankfurt, Germany; 3 German Cancer Consortium (DKTK), Partner Site Frankfurt/Mainz, a partnership between DKFZ and University Medicine Frankfurt, Frankfurt am Main, Germany

**Keywords:** ATC code, Dictionary, R-tool, Standardizing free-text, Substance dictionary, Medication dictionary, Language processing, Cancer therapy, Data processing

## Abstract

Over the past decades, oncology treatment paradigms have developed significantly. Yet, the often unstructured nature of substance-related documentation in medical records presents a time-consuming challenge for analyzing treatment patterns and outcomes. To advance oncological research further, clinical data science must offer solutions that facilitate research and analysis with real-world data (RWD). The present contribution introduces a user-friendly R-tool designed to transform free-text medication entries into the structured Anatomical Therapeutic Chemical (ATC) Classification System by applying a dictionary-based approach. The resulting output is a structured data frame containing columns for antineoplastic medication, other medications, and supplementary information. For accuracy validation, 561 data entries from an evaluation data set were reviewed, consisting of 935 tokens. 88.5% of these tokens were successfully transformed into their respective ATC codes. Additional information was extracted from 129 data entries (23%), while 23 entries (4.1%) presented no usable information. All tokens underwent a manual review; 8.9% (84 tokens) failed transformations. This approach improves the standardization and analysis of systemic anti-cancer treatment data in German-speaking regions by optimizing efficiency while maintaining relevant accuracy.

## Introduction

**Fig. 1:**
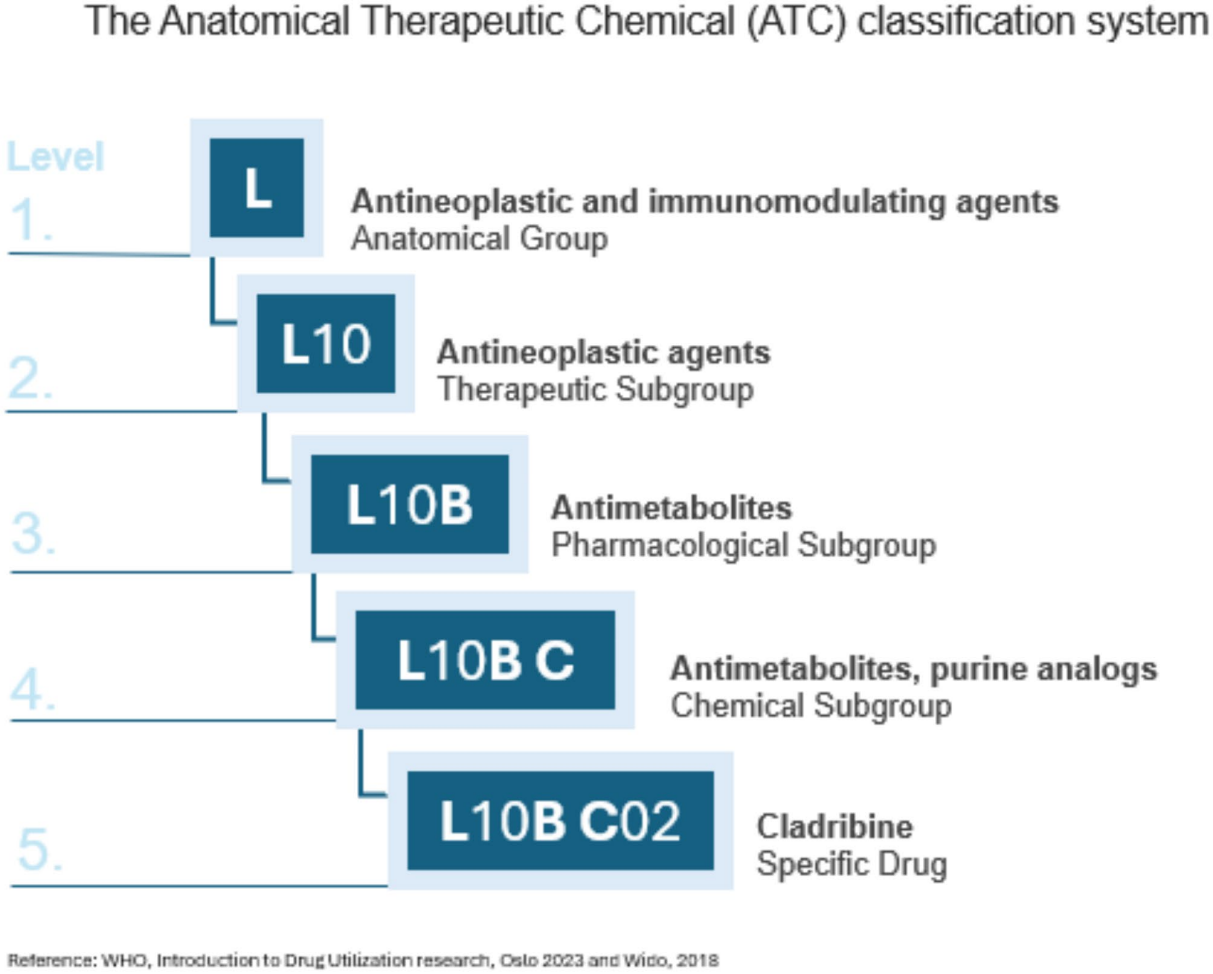
Structure of ATC code and dictionary^6,8^.

Over the past decades, substantial innovations have transformed oncological treatment and significantly optimized patient outcomes^1^. However, the unique characteristics of diseases and individual patients result in heterogeneous treatment options^2^. This complexity is further increased by the evolution of therapies throughout the patient’s disease course and advancements in research^3^. Consequently, treatment regimens can vary between patients and over time, especially in the context of second- or third-line therapies^4^. In medical records, treatment details are often recorded in unstructured formats, such as free-text or custom-made catalogs. This lack of format and thus standardization poses challenges for researchers aiming to analyze therapy patterns regarding, e.g., outcomes^5^. In turn, implementing a structured format can save resources and enable a more thorough analysis of relevant research questions. Recognizing the need for a secure and structured approach, an R-tool was developed using a dictionary-based approach to transform free-text medication entries into Anatomical Therapeutic Chemical (ATC) codes^6^. The ATC system provides a universal and standardized medication classification, organizing them into five hierarchical levels (Fig. 1). The first level categorizes medications into fourteen anatomical groups based on the targeted organ or anatomical system. Medications are further divided across three levels of substance subgroups, with the fifth level (consisting of one letter, two numbers, two letters, and two numbers) representing the specific medication code. The dictionary-based approach requires minimal computational resources and is easily distributable, deterministic, reliable, and customizable. Additionally, R is a widely used and accessible programming language in data science, making it a practical choice for researchers^7^.

## Methods

### Tool development

**Fig. 2:**
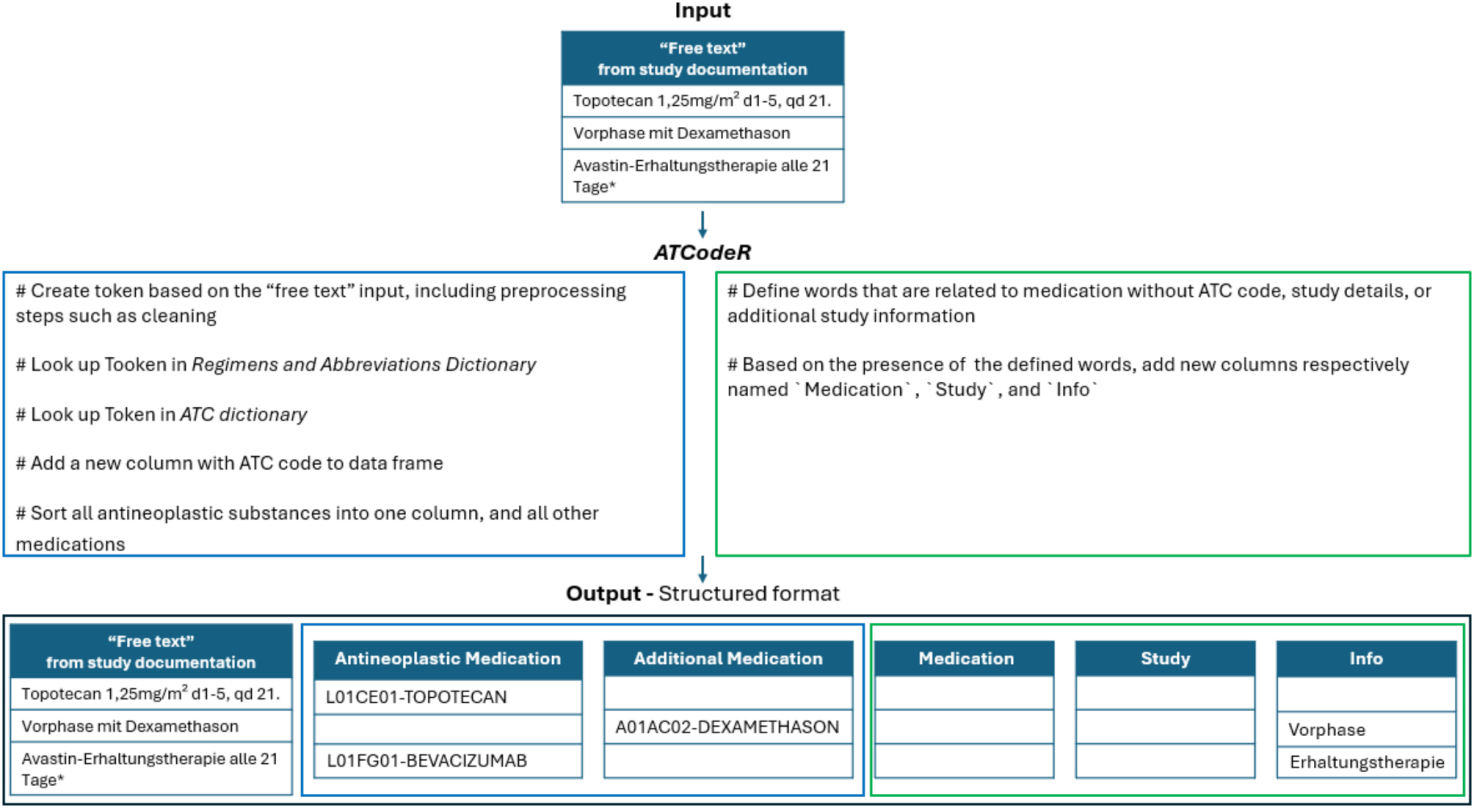
The figure displays the input, *ATCodeR* function, and output structure for three example data entries. The function provides only a summary and is not complete.

We developed an R-tool called *ATCodeR,* designed to standardize medication data into ATC codes. Figure 2 illustrates the functionality of *ATCodeR*.

*Input*: The input is a single-column data frame with free-text descriptions of administered medication. Each row might, e.g., represents a medication administration event for an individual patient.

*Output*: The output is a data frame retaining the original input column of free-text medication and adds five new columns:

1. *AntineoplasticMedication:* Contains ATC codes for antineoplastic medications, i.e., all chapter *L* codes in the hierarchical ATC structure (Fig. 1).
2. *AdditionalMedication:* Contains codes that start with any letter other than *L*.
3. *Medication:* Lists all other substances identified as medication names but lacking an associated ATC code.
4. *Study:* Indicates whether the medication was administered as a part of a clinical trial.
5. *Info:* Provides supplementary therapy details, such as “TACE” for “Transarterial chemoembolization”.

The next sections encompass the entire *ATCodeR* processing functionality, starting from the following “Medication name preprocessing” through to the “Function output”.

### Medication names preprocessing

Development: As the R-tool was developed for use within the German Cancer Consortium’s (DKTK) Clinical Communication Platform, it was designed to handle the specific characteristics of German medication names. For development purposes, a comprehensive test data set (TDS) was generated.

Application: In the very first step of the *ATCodeR* application for any data set, German umlauts and special characters (ä, ö, ü, ß) are replaced with “ae”, oe”, “ue”, and “ss”, respectively.

Subsequently, free-text medication entries are converted into so-called tokens (i.e., character strings that represent entity terms or, in this case, a single medication or regimen), with punctuation, symbols, and hyphens removed simultaneously using the quanteda package’s^9^ *tokens* function. Undesired tokens (e.g., measurement units, time references, and combinations of letters and numbers) are removed by applying a manually curated list using the *quanteda::tokens_remove* function. This is performed because the input data set is likely to be heterogeneously annotated with contextual information such as dosage and administration route unavailable in most data entries.

Finally, all remaining tokens are converted to uppercase by *quanteda::tokens_toupper*.

### Medication transformation step 1: regimens and abbreviations dictionary

Development: We developed a regimens and abbreviations dictionary (RAD) by systematically scanning the TDS and its free-text medication entries for standard regimens and other abbreviations. Respective entries to the RAD were organized alphabetically in a nested list and subsequently converted into a dictionary using the *quanteda::dictionary* function.

Application: The generated tokens from the preprocessed data input are matched against the RAD using the *quanteda::tokens_lookup* function.

Single medication entries that are converted to two tokens (e.g., input “5-FU (Fluorouracil)” are matched to “Fluorouracil Fluorouracil”) are deduplicated.

### Medication transformation step 2: ATC dictionary

Development: For this step, we employed the ATC dictionary, sourced from WIdO [Wissenschaftliches Institut der AOK [Allgemeine Ortskrankenkassen], English: AOK Research Institute^8^]. We imported the ATC data into a Microsoft Excel^10^ spreadsheet. We organized the WIdO-data into a nested list for the dictionary construction.

We used *quanteda::dictionary* to convert the nested list into a dictionary object and applied it to the TDS. For refinement, we reviewed untransformed tokens of the TDS after initial application. These tokens were either categorized as “irrelevant words” or added to the RAD or ATC Dictionary.

Application: Tokens of free-text medication in the input data set are transformed into standardized ATC codes by using *quanteda::tokens_lookup* and are sorted alphabetically, and added as a new column to the original data frame.

### Function output

Development: We conducted multiple rounds of extensive refinement to finalize and optimize the transformation outcomes, including identifying and correcting falsely transformed tokens as previously described.

Application: In the resulting data frame, antineoplastic medications are separated from other medications and organized into distinct columns (see Fig. 2). If a medication is associated with multiple ATC codes (e.g., Methotrexate being transformed to both L01BA01 and M01CX0), only the ATC code starting with *L* is retained. This rule was chosen due to the R-tool’s primary focus on cancer therapies. In cases where no *L*-ATC code is available among multiple transformations for a single medication, the first transformation available in alphabetical order is selected. As the data set is unlikely to provide any information on administration routes and dosage, this rule was applied to ensure a systematic and standardized approach. Three additional columns are established. The first represents medications without an ATC code (“*Medication*”), a second that comprises study-related information (“*Studie*”, i.e., indicating whether the therapy was part of a clinical study), and a third that displays supplementary therapy specifications (“*Info*”, e.g., Erhaltungstherapie (English: Maintenance therapy). These new columns, along with the initial free-text medication entry column, comprise the final data frame.

### Evaluation study

For evaluation, the *ATCodeR* was applied to a sample of 561 unique data entries sourced from the University Cancer Center Frankfurt, covering medications for solid cancer entities. The data entries used for the evaluation study differed from the TDS used to develop the R-tool to ensure an independent assessment. The output was manually reviewed by two authors to verify the accuracy of the classification for evaluation purposes. In the first step, we identified all tokens per entry expected to be transformed into an ATC code as “ATC code expected”. The result was compared to the actual output presented in columns *AntineoplasticMedication* and *AdditionalMedication*. If an expected transformation was missing in either one of the columns, an “Error in transformation” was indicated. For completeness, identified medication names were further incorporated into the RAD. An entry that did not provide an ATC code, additional information (in column *Medication*, *Study* or *Info*), or was assumed to be an “Error in transformation” was categorized as “No useful information”.

Additionally, we also roughly estimated the time required for manually transforming tree-text medication into ATC codes. This process involved measuring the time of copying the medication name, searching for it using a search engine, selecting the appropriate ATC code, and, if the provided name was a regimen, also identifying the medication name(s) and then copying the respective ATC code(s) and name(s) back into a table.

## Results

### Evaluation results

**Fig. 3:**
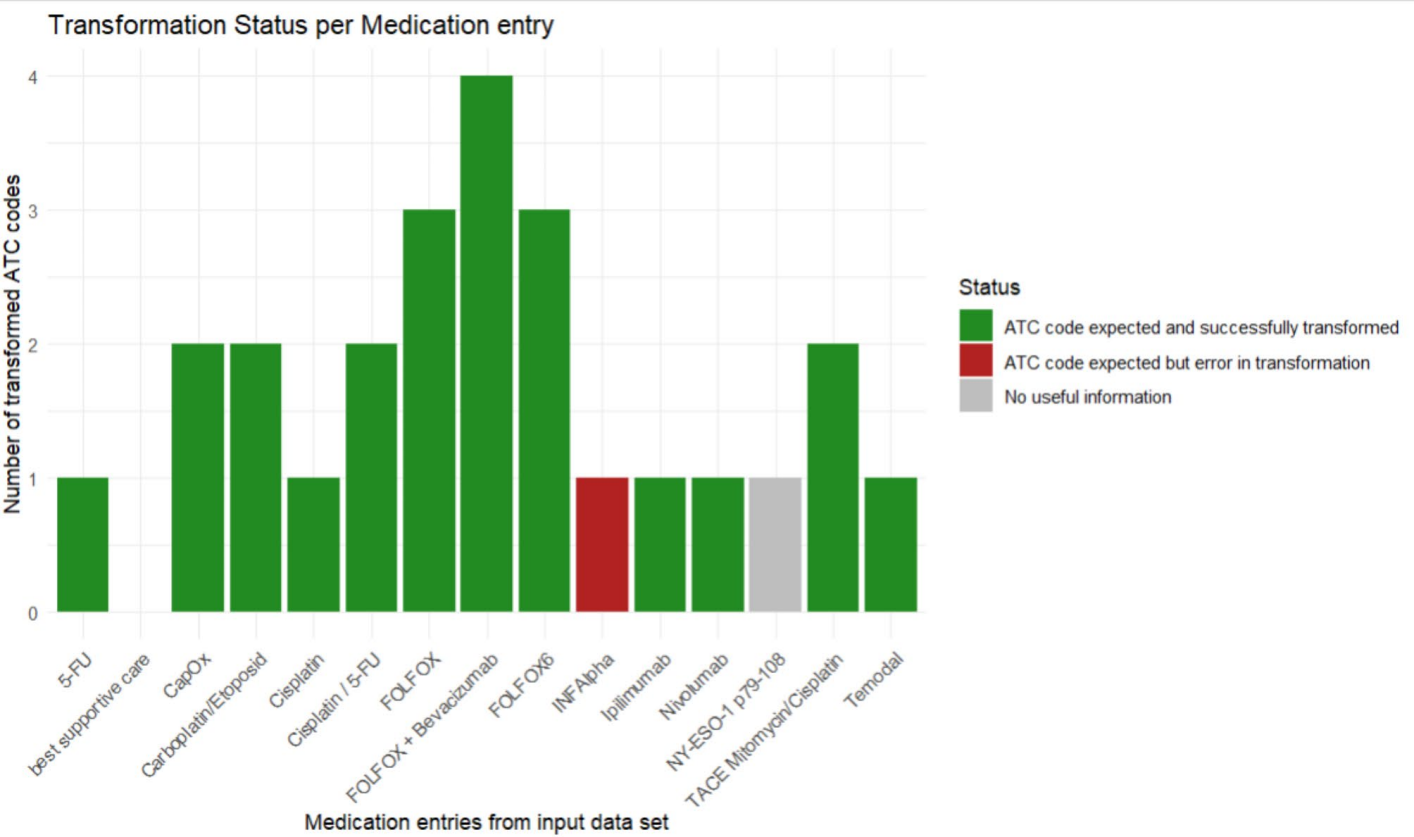
Subset of the *ATCodeR* evaluation output. 15 medication entries are displayed based on the evaluation input data frame. No bar is shown for “best supportive care” as it was not expected to be transformed into an ATC code (though it was added to the *“Info”* column).

A total of 561 data entries, including 1359 tokens (ranging from one to four tokens per entry) were examined from the evaluation data set. A subset overview of the *ATCodeR* evaluation output is provided in Fig. 3.

We found 88.5% (n = 828) of the identified “ATC code expected” tokens (n = 935) to be transformed into respective ATC codes. Our evaluation further revealed 72 data entries with “Error in Transformation”, referring to a total of 84 medication tokens. As expected, unknown, incomplete, or residual abbreviations (e.g., “Mitomyc”, “Ipi”, “Nevo”, “Cisplat”) represented the main reason for missing transformation into ATC codes, followed by typographical errors. A respective overview can be found in Table 1. Since this data set included information from patients that were routinely treated at the University Cancer Center Frankfurt, as well as patients enrolled in clinical trials, the data set also contained study medications not yet registered with an ATC code and study registry names. Respective terms presented challenges during transformation, as they could not be linked to standardized ATC codes or additional information. These entries were marked as “No useful information”. In total, 23 entries (4.1%, each entry counted as one token) presented similar medication or study terms and were thus flagged as “No useful information”.

**Table 1:** Overview of evaluation results.

	Overall	Successfully transformed	Failed to transform	No useful information
Data entries	561 (100%)	466 (83%)	72 (12.9%)	23 (4.1%)
Tokens	935 (100%)	828 (88.5%)	84 (8.9%)	23 (2.6%)

The first row displays outcomes at the data entry level, while the second row reflects token-level performance.

In the evaluation output data frame, additional information (from the *Study* and *Info* columns) was extracted from 129 entries (23%), displaying treatment details, such as “Studie” (English: study), “Register” (English: registry), “Protokoll” (English: protocol), “TACE” (Transcatheter arterial chemoembolization), “Chemotherapie” (English: chemotherapy), or “Erhaltungstherapie (English: maintenance therapy). No additional information was recorded in the *Medication* column.

Looking up medications manually in a medication register typically took around 30 s per medication. With 983 tokens, this process roughly takes 8 h of human effort. In contrast, *ATCodeR* completed the transformation in less than 10 s, demonstrating a substantial improvement in efficiency.

## Discussion

Our R-tool exemplifies a solution specifically crafted to streamline tasks that often rely on labor- and time-intensive manual processes. Hence, *ATCodeR* enhances the analysis of research questions related to therapeutic plans or regimens.

The evaluation revealed that the majority of medications were accurately mapped to ATC codes. Considering the nature of the data entries, the evaluation result (88.5% accuracy in transformation) can be considered satisfactory. We should emphasize that the evaluation data set consisted of study-specific entries originating from a single cancer center-based registration unit (University Cancer Center Frankfurt) and that each of the 561 entries was unique, only appearing once in the data set. In research contexts where the R-tool is applied to regular patient cohorts, two notable differences can be expected. In these cohorts, the percentage of standard treatments is typically much higher, and individual medication entries are likely to occur multiple times. Both of these factors could lead to even greater benefits and accuracy than demonstrated in the evaluation study.

To contextualize our R-tool within the landscape of publicly available solutions, one relevant tool is the DiAna dictionary, an open-source resource for standardizing free-text medication names. In a recent study^11^, DiAna successfully mapped 346,854 complex entries to their respective ATC codes, achieving a 98.94% standardization rate and demonstrating high accuracy. While DiAna is designed for large-scale pharmacovigilance data sets, our R-tool is tailored to efficiently transform German free-text medication entries into ATC codes within a research-focused contexts. Our R-tool further includes a specific regimens and abbreviations dictionary (RAD), a key feature in the context of cancer treatment. Notably, Fusaroli et al.^11^ encountered similar challenges to those identified in our evaluation, highlighting that respective issues are common in standardization efforts. Like our approach, they also faced difficulties when multiple ATC codes were available for a single medication, which they faced by establishing specific rules and, in some cases, even manually adjusting transformations.

In light of the challenges associated with transforming medication tokens containing typographical errors, misspellings, or incomplete abbreviations, the *ATCodeR*’s high degree of customizability presents a significant advantage. Common misspellings or abbreviations from a new input data set can easily be added to the RAD, enabling the R-tool to adapt seamlessly to various requirements and contexts.

To broaden the scope of the R-tool, its standardized format offers the potential to extend similar strategies for standardizing other formats of medical data. To illustrate, the R-tool could be expanded by integrating supplementary RADs, making it applicable to a wider range of medication investigations, e.g., on antibiotic treatments. Accordingly, the R-tool could enable researchers to analyze (longitudinal) treatment choices and modifications more effectively, thereby allowing to generate and test hypotheses regarding treatment efficacy, safety, patient outcomes, and overall management. However, incorporating patient identification in terms of pseudonymization will become crucial for accurate analysis and tracking. Compared to artificial intelligence, including large language models (LLM) [e.g., OpenAI’s ChatGPT^12^], custom-built research solutions like *ATCodeR* are likely better suited to navigate the complexities of healthcare data use, as the current German data regulatory landscape holds substantial challenges for integrating third-party entities into medical data workflows^13^. However, another potential application of LLMs within the context of the R-tool could be the completion of failed or falsely identified ATC codes via an Application Programming Interface (API), integrated into the R environment. We opted not to implement this feature due to the associated API usage costs.

In conclusion, the present approach aligns with broader efforts to optimize healthcare through digital solutions and the harmonization of RWD, ultimately aiming to improve clinical outcomes and operational efficiency.

### Limitations

One main limitation of the ATCodeR is its current restriction to processing exclusively German free-text inputs. Ideally, the tool’s capability could be expanded in the future to accommodate multiple languages, thereby broadening its applicability. Another notable limitation is the tool’s inability to handle unknown misspellings or typographical errors. Although medical records are expected to be error-free, simple errors, such as the transposition of single letters, frequently occur during data entry. Such unrecognized errors can potentially affect the accuracy of the tool’s output. Furthermore, the development and testing of the R-tool at a single center present an additional limitation. If implemented at other sites with different documentation standards, the transformation of medications may be less accurate. Therefore, multi-center studies are necessary to enhance the dictionary’s comprehensiveness and improve the tool’s accuracy.

## Conclusion

In conclusion, the presented R-tool *ATCodeR* significantly enhances the standardization of oncology treatment data in German-speaking regions. By offering an easy-to-apply and customizable tool, it addresses data processing challenges and enables more efficient research workflows. Therefore, this R-tool has the potential to streamline research efforts and ultimately lead to advancements in oncology through improved data consistency.

## Data Availability

The R script as an R package is publicly available on GitHub: https://github.com/IsiSchnorr/ATCodeR

## Abbreviations

ATC: Anatomical Therapeutic Chemical
AOK: Allgemeine Ortskrankenkassen
DKTK: German: Deutsches Konsortium für Translationale Krebsforschung; English: German Cancer Consortium
TACE: Transarterial chemoembolization
RWD: Real-world data
WidO: German: Wissenschaftliches Institut der AOK, English: AOK Research Institute
